# Atorvastatin treatment and LDL cholesterol target attainment in patients at very high cardiovascular risk

**DOI:** 10.1007/s00392-016-0991-z

**Published:** 2016-04-27

**Authors:** Ulrich Laufs, Barbara Karmann, David Pittrow

**Affiliations:** 1 Innere Medizin III-Kardiologie, Angiologie und internistische Intensivmedizin, Universitätsklinikum des Saarlandes und Medizinische Fakultät der Universität des Saarlandes, Kirrberger Straße 100, 66424 Homburg, Germany; 2 Medical Department, MSD SHARP & DOHME GmbH, Haar, Germany; 3 Institut für Klinische Pharmakologie, Medizinische Fakultät, Technische Universität Dresden, Dresden, Germany

**Keywords:** LDL cholesterol, Atorvastatin, Pharmacoepidemiology, Statins, Management, Cardiovascular risk

## Abstract

The use of atorvastatin is rapidly increasing among statins since the introduction of generics. However, only limited data are available on its current use and the effectiveness outside of randomised trials. The aim of the study was to assess low-density lipoprotein (LDL-C) levels in ambulatory patients at very high cardiovascular risk on atorvastatin therapy in physician’s offices. A total of 2625 high-risk patients on atorvastatin were included into this cross-sectional study by 539 office-based physicians between June and December 2014. 47.0 % of the patients had documented coronary heart disease (CHD), 25.1 % type 2 diabetes mellitus (DM), and 27.9 % CHD plus concomitant DM. The mean age was 66.1 ± 10.8 years, 62.1 % were male. Atorvastatin at the dose of 10, 20, 40 and 80 mg/day was administered in 15.6, 45.7, 33.9, and 4.8 % of the patients, respectively. The treatment duration was 92.6 ± 109.6 weeks. The mean atorvastatin dose at therapy start was 24.8 ± 15.2 mg/day and at time of documentation 27.9 ± 15.8 mg/day. Low-density lipoprotein cholesterol (LDL-C) <70 mg/dL was achieved by 10.5 % of the total cohort (7.5 % in DM, 9.3 % in CHD, and 15.2 % in CHD + DM). In contrast, according to physicians’ subjective assessment, 62.7 % of patients (with small differences between groups) had reached their individual LDL-C target. In summary, higher doses of atorvastatin are not frequently used in clinical practice. The LDL-C target level <70 mg/dL as recommended by current guidelines is achieved only in a minority of atorvastatin treated patients at very high cardiovascular risk.

## Background

Low-density lipoprotein cholesterol (LDL-C) serum concentrations correlate with cardiovascular (CV) risk and lowering of LDL-C reduces CV events. Based on many large randomized clinical studies, LDL-C lowering with statins is recommended for the secondary prevention of cardiovascular outcomes [5, 33, 36]. The current European guidelines (issued 2011 by EAS/ESC) [33] as well as the US guidelines [36] (issued 2013 by AHA/ACC) on the treatment of hyperlipidaemia pose particular emphasis on patients with very high cardiovascular risk and recommend stringent LDL-C lowering with statins. While the US guidelines recommend fixed high doses (e.g,. atorvastatin 80 mg) for high-risk individuals [36], the European guidelines recommend a treatment goal of LDL-C <70 mg/dL or a >50 % LDL-C reduction [33].

According to a recent German study in primary care, the prevalence of diagnosed coronary heart disease (CHD) is 12 %, and of diabetes mellitus type 2 (DM) 15 % [31]. Both diseases often present concomitantly [31]. Patients with either CHD or DM (and those with stroke, peripheral arterial disease or chronic renal insufficiency) are at very high risk and can especially benefit from lipid lowering [33].

The utilization of statins in Germany increased steadily in the recent years, up to 1707 million daily doses in 2013 for all statins combined [17]. Because of its designation as lead substance within this group in 2006, simvastatin was most widely prescribed in the past. However, since introduction of atorvastatin as generic drug in March 2013, for this agent 250 million daily doses were administered, with further increase anticipated [17]. Among the generic statins, atorvastatin is perceived as the most potent agent and atorvastatin will likely be the most frequently prescribed statin in the near future [13, 20, 27].

Despite the increasing use of atorvastatin, current information on the drug under clinical practice conditions is limited compared to evidence on simvastatin in this setting [11, 37] Against this background, the cross-sectional study DISCOVER was initiated to assess the standard of care in ambulatory patients at very high cardiovascular risk, treated with atorvastatin monotherapy (original drug or generics).

## Methods

DISCOVER was performed as retrospective, cross-sectional study in the offices of 539 physicians in all regions of Germany between June and December 2014. Study materials were approved by the ethics committee of the Bavarian Physicians Chamber in Munich on 8 May 2014. DISCOVER was registered in the vfa study database (No. 931). Cardiologists, diabetologists, internists and general practitioners agreed to enter data of up to first five eligible patients into the electronic case collection form.

Patients were eligible for documentation if they met the following criteria:

- ≥18 years of age at the documentation visit;
- diagnosed CHD, diagnosed DM, or both conditions concomitantly (CHD + DM);
- current treatment with atorvastatin, stable for at least 1 month;
- LDL-C laboratory value available.

Patients on other lipid-lowering therapies were not eligible. The following data were collected: age, gender, weight, height, and waist circumference, health insurance status (statutory or private), index diagnoses (DM, CHD), smoking, arterial hypertension, cardiovascular events in the patient history (myocardial infarction, stroke, and transient ischaemic attack), previous cardiac interventions [e.g., percutaneous coronary intervention (PCI), coronary artery bypass grafting (CABG)].

Atorvastatin therapy was documented with the current daily dose (mg/day), the treatment duration and the daily dose at the start of the treatment. Concomitant medication for the treatment of CHD and DM was also recorded. Further information on other statins or other lipid-lowering drugs used in the past was documented.

Systolic and diastolic blood pressure values were recorded, as were laboratory values to assess blood fasting glucose and HbA_1c_ in diabetic patients, and lipid values (total cholesterol, LDL-C, HDL-C, triglycerides) in all patients. Physicians assessed the lipid-lowering target achievement on atorvastatin treatment (yes/no), and the satisfaction with current lipid-lowering treatment with atorvastatin (yes/no).

A quality management system was implemented and standard operating procedures (SOPs) were used to ensure that the study was conducted and data were generated, documented, and reported in compliance with the protocol.

Upon submission of data, each investigator verified all recorded data to be accurate. Additionally, queries were generated by data management during and after the study to resolve any questions and implausible data. Implausible data that could not be clarified were deleted from the database. For the analysis, missing values were not replaced. The entries “unknown” and “not collected” were treated like missing values.

### Statistical methods

Categorical variables were shown as absolute and adjusted relative frequencies including the number of missing values in each category. Continuous variables were to be presented as means with standard deviation, median, quartiles, minimum value and maximum value, and number of known values. The statistical analysis system (SAS) software package, release 9.2 (Cary, NC, U.S.A.) was used.

## Results

### Physicians

A total of 539 physicians contributed to the study. The majority (*n* = 418; 78 %) documented five patients. Of the 2625 patients included, 1699 visited the office of a general practitioner, 687 an internist, 175 patients a cardiologist, and 221 a diabetologist (some physicians had more than one specialization).

### Characteristics

Characteristics are shown in Table 1. 1233 patients had CHD (47.0 %), 658 patients DM (25.1 %), and 734 CHD plus DM (27.9 %). The mean age of the patients was 66.1 ± 10.8 years (range 19.0–93.0 years). Males were more frequently included than females (62.1 versus 37.9 %). The majority of patients were in the statutory health insurance (89.6 %), while a smaller share had private insurance (10.4 %).

**Table 1:** Demographic characteristics, comorbidities and risk factors

Parameter	Subgroup				*P* value
	Total *N* = 2625	CHD *N* = 1233	DM *N* = 658	CHD + DM *N* = 734	
Age (years)	*n* = 2622	*n* = 1232	*n* = 658	*n* = 732	<0.001^a^
Mean ± SD	66.1 ± 10.8	65.8 ± 11.0	63.8 ± 10.9	68.7 ± 9.9	
Range	19.0–93.0	19.0–93.0	33.0–92.0	36.0–93.0	
Sex, *n* (%)	*n* = 2625	*n* = 1233	*n* = 658	*n* = 734	<0.001^b^
Male	1630 (62.1)	818 (66.3)	329 (50.0)	483 (65.8)	
Female	995 (37.9)	415 (33.7)	329 (50.0)	251 (34.2)	
Body mass index (kg/m^2)^	2422	1108	617	697	<0.001^a^
Mean ± SD	28.9 ± 4.7	27.5 ± 3.9	30.1 ± 5.4	29.9 ± 4.7	
Arterial hypertension, *n* (%)	2198 (86.6)	987 (83.7)	526 (82.6)	685 (94.7)	<0.001^a^
Family history of CHD, *n* (%)	1073 (60.7 %)	542 (63.6)	202 (45.7)	329 (69.6)	
Previous MI, *n* (%)	853 (34.1)	487 (41.9)	21 (3.4)	345 (48.3)	
Previous PCI, *n* (%)	900 (36.3)	547 (47.5)	13 (2.1)	340 (48.3)	
Previous CABG, *n* (%)	351 (14.0)	203 (17.4)	10 (1.6)	138 (19.4)	
Microalbuminuria, *n* (%)	311 (14.2)	23 (2.4)	108 (18.5)	180 (27.5)	<0.001^a^
Previous stroke, *n* (%)	159 (6.4)	68 (5.9)	35 (5.6)	56 (7.9)	<0.001^a^
Previous TIA, *n* (%)	180 (7.2)	83 (7.2)	26 (4.2)	71 (10.0)	<0.001^a^

Values are *n* (%) if not stated otherwise

*CABG* coronary artery bypass graft, *CHD* coronary heart disease, *DM* diabetes mellitus, *SD* standard deviation

*P* values were calculated by ^a^ Kruskal–Wallis test or ^b^ Chi-square test

### Treatment

Atorvastatin was administered in a wide dosing range up to 80 mg/day. The mean dose at therapy initiation was 24.8 ± 15.2 mg/day, and at the time of documentation 27.9 ± 15.8 mg/day, with no major differences between the groups (CHD 28.6 ± 16.3 mg/day, DM 24.6 ± 13.3 mg/day, CHD + DM 29.8 ± 16.4 mg/day). The 10, 20, 40 and 80 mg/day atorvastatin doses were administered in 15.6, 45.7, 33.9, and 4.8 % of the patients, respectively, without relevant differences across subgroups. Mean treatment duration was 92.6 ± 109.6 weeks (range 4–886 weeks) before the documentation date, with no major differences between treatment groups. There was no association between the atorvastatin dose (neither at initiation of therapy or at documentation) and the treatment duration.

### Laboratory values and blood pressure

The mean value for LDL-C was 116.3 ± 42.6 mg/dl, for total cholesterol 194.5 ± 50.6 mg/dl, for HDL-C 52.2 ± 17.7 mg/dl, and for triglycerides 172.4 ± 101.0 mg/dl. As shown in Table 2, TC and LDL-C were somewhat lower in the CHD + DM group, HDL-C was higher and triglycerides were lower in the CHD group. Patients in the DM group exhibited higher TC, LDL-C, and triglyceride values compared to the other groups.

**Table 2:** Lipid values, blood pressure and blood glucose

Measurement	Subgroup				*P* value
	Total *N* = 2625	CHD *N* = 1233	DM *N* = 658	CHD + DM *N* = 734	
Total cholesterol, mg/dL	*n* = 2499	*n* = 1157	*n* = 631	*n* = 711	<0.001
	194.6 ± 50.6	194.1 ± 50.1	203.4 ± 50.7	187.5 ± 50.1	
LDL-C, mg/dL	*n* = 2612	*n* = 1227	*n* = 653	*n* = 732	<0.001
	116.3 ± 42.6	115.8 ± 41.0	123.9 ± 44.4	110.2 ± 42.6	
HDL-C, mg/dL	*n* = 2416	*n* = 1120	*n* = 607	*n* = 689	<0.001
	52.2 ± 17.7	54.8 ± 19.2	51.8 ± 16.6	48.4 ± 15.0	
Triglycerides, mg/dL	*n* = 2392	*n* = 1104	*n* = 602	*n* = 686	<0.001
	172.4 ± 101.0	150.6 ± 78.3	193.1 ± 117.3	189.5 ± 110.5	
Systolic blood pressure, mmHg	*n* = 2503	*n* = 1161	*n* = 629	*n* = 713	<0.001
	133.3 ± 13.3	131.5 ± 13.3	135.1 ± 12.9	134.7 ± 13.4	
Diastolic blood pressure, mmHg	*n* = 2497	*n* = 1160	*n* = 627	*n* = 710	<0.001
	79.6 ± 8.4	79.0 ± 8.2	80.7 ± 8.6	79.5 ± 8.6	
HbA1c, %	*n* = 1658	*n* = 346	*n* = 620	*n* = 692	<0.001
	6.6 ± 1.4	5.2 ± 1.8	7.0 ± 1.0	7.0 ± 1.0	
Fasting glucose, mg/dL	*n* = 2040	*n* = 799	*n* = 574	*n* = 667	<0.001
	115.4 ± 37.9	91.6 ± 19.7	127.5 ± 38.1	133.5 ± 39.3	

All values are mean ± standard deviation, if not indicated otherwise

*P* values were calculated by Kruskal–Wallis test

*CHD* coronary heart disease, *DM* diabetes mellitus

Mean systolic blood pressure was 133.3 ± 13.3 mmHg, and diastolic blood pressure 79.6 ± 8.4 mmHg. The mean systolic value was lower in the CHD group compared to the other subgroups, while the mean diastolic values were similar across groups.

### LDL-C target level achievement

**Fig. 1:**
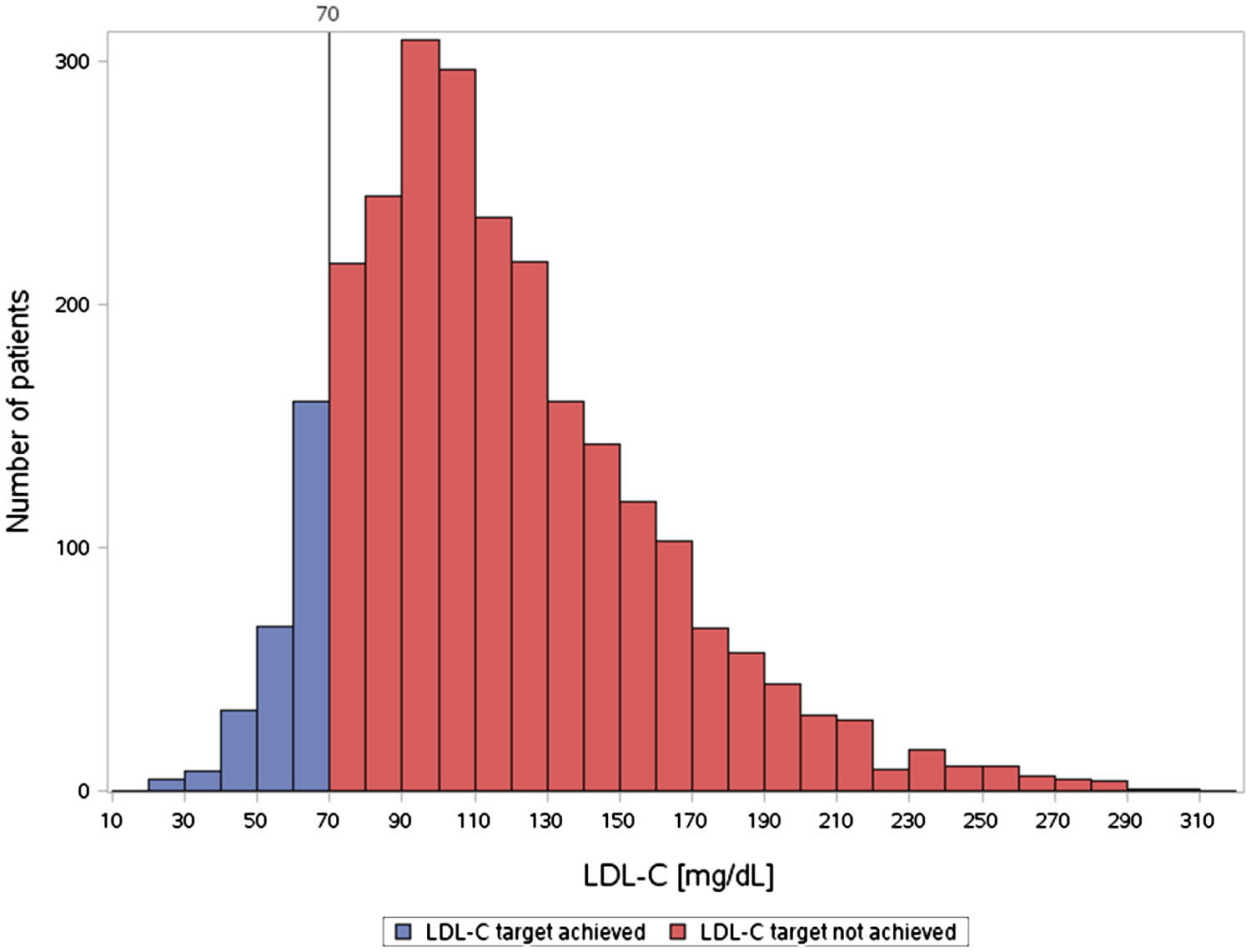
Histogram of LDL-C categories at the documentation visit. *P* < 0.001 (Chi-square test) each for the comparison of the CHD, DM and CHD + DM groups with respect to LDL-C <70 mg/dL, or with respect to treating physician’s assessment

**Fig. 2:**
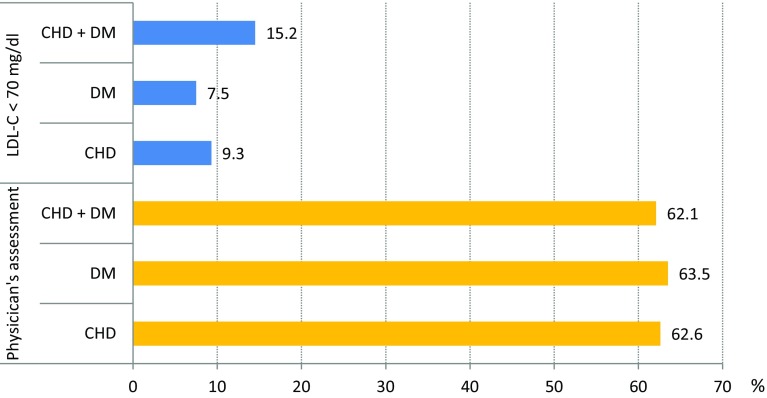
LDL-C target attainment by laboratory results (<70 mg/dL) and subjective physician assessment. *Figure* shows the percentages of patients with LDL-C <70 mg/dl (*blue bars*) and in comparison the percentages of patients that were judged by their physicians to have clinically met their individual LDL-C target

The distribution of LDL-C values in the total cohort is shown in Fig. 1. The LDL-C target value of <70 mg/dL was achieved only by 10.5 % of the patients overall. The rate was lowest in the DM group (7.5 %) and somewhat higher in the CHD group (9.3 %) and the DM + CHD group (15.2 %), respectively (Fig. 2 top).

Target level achievement rates were not associated with the duration of atorvastatin treatment. Patients who met the LDL-C target compared to those who did not had similar treatment duration (93.1 ± 96.3 versus 92.7 ± 111.3 weeks).

**Fig. 3:**
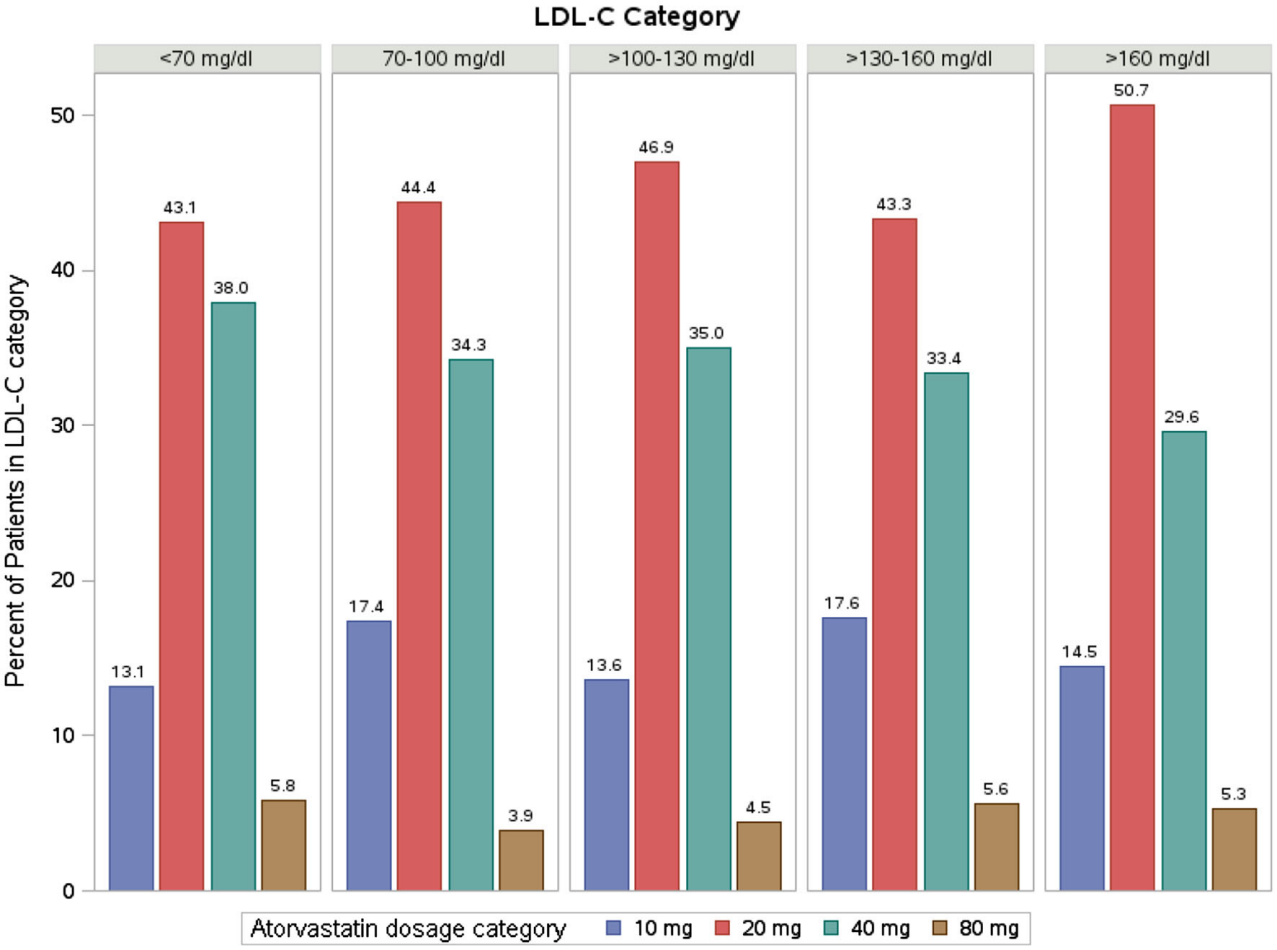
Distribution of atorvastatin dosages by LDL-C category. The distribution of atorvastatin dosage categories (mg/day) was similar across all LDL-C categories

The distribution of atorvastatin dosage categories (mg/day) was similar across all LDL-C categories (Fig. 3).

The subjective physician assessment of target achievement substantially deviated from the laboratory values. While 10.5 % of patients who had not achieved the target according to the LDL-C criterion (<70 mg/dL), 62.7 % were assessed by their physicians to have clinically met the target (Fig. 2 bottom).

Overall, 74.3 % of physicians were satisfied with the atorvastatin therapy, with marginal differences across subgroups. However, satisfaction decreased with increasing atorvastatin dose: 78.8 % of physicians were satisfied with the 10 mg/day dose in contrast to 63.2 % with the 80 mg/day dose.

## Discussion

The two main findings of this contemporary cross-sectional analysis are that patients at very high cardiovascular risk treated with atorvastatin receive relatively low doses and that only a minority of the patients achieved an LDC-C below <70 mg/dL.

Lipid abnormalities are highly prevalent under clinical practice conditions [6, 9, 10, 12, 37]. Despite the documented benefit of statin therapy and the consensus of the guidelines on intensive treatment for high-risk patients, the standard of care documented in this large contemporary sample reveals a substantially different situation. The incomplete implementation of guideline recommendations supports data from US office-based cardiologists who did not use statins in 32.4 % of those patients who were eligible for such medication [21]. In other countries similar findings were reported [34, 35]. In the EUROASPIRE IV study in 76 centers of 24 European countries an improved situation in the management of CHD patients was shown: at least on the short term statins were administered to 85.7 % of eligible patients (with or without other cardioprotective medications) [18]. This represents a clear improvement to an earlier study in Germany, in which the treatment rates with statins (and other cardioprotective medications) decreased substantially over time: after 5 years only 17 % of eligible patients, who originally had received statins, were still on such agents [22]. Our study now provides evidence that even in the patients that receive a potent statin, a majority of patients receives a low dose.

Atorvastatin, introduced in 1996, is a well-studied statin with high potency to lower elevated LDL-C levels. It is prescribed to a large portion of statin-treated patients around the world. Data from randomized trials suggest that the full dose of 80 mg atorvastatin can lower LDL-C by 45–55 % [19, 25, 29]. Indeed, for atorvastatin the “fire and forget strategy” was propagated based on the assumption owing to its strong LDL-C lowering effect the drug would not require follow-up examination [4]. However, this assumption only applies for the drug given at high doses [33]. In an analysis of the VOYAGER database, 40 or 59 % of patients on atorvastatin 40 or 80 mg/day achieved more than 50 % decrease of LDL-C [15]. This strong lowering of LDL-C by atorvastatin was associated with a reduction of major coronary events across all investigated risk groups [26].

To our knowledge, current observational data on atorvastatin use and effectiveness in clinical practice is limited to two reports. In the UK, of 2999 high-risk patients (60.2 % men; mean age 67.9 ± 10.6 years), 23.9, 28.2, 36.2, and 11.6 % were administered atorvastatin 10, 20, 40, and 80 mg, respectively [14]. Across all doses, the mean LDL-C level was 81 ± 27 mg/dL, and 46.5 % had LDL-C <77 mg/dL [14]. In an analysis of chart reviews of two health insurance databases, 21.8, 29.6, 29.9, and 18.7 % (GE Centricity EMR) and 25.4, 32.9, 27.8, and 14.0 % (Humana Medicare) received 10, 20, 40, and 80 mg/day doses of atorvastatin, respectively [24]. The mean follow-up LDL-C levels were 83 ± 30 and 88 ± 31 mg/dL for the GE Centricity EMR and Humana Medicare cohorts, respectively. Regardless of dose, only 28.3–34.8 % of patients had LDL-C <70 mg/dL [24].

Our data show a mean LDL-C level of 116.3 mg/dL. Importantly, only 10.5 % of patients reached LDL-C values below 70 mg/dL. These findings confirm earlier reports from clinical practice suggesting that physicians are reluctant to administer high atorvastatin doses. Registries such as DYSIS [2] and 2L [11] report simvastatin doses in the lower or intermediate range. DISCOVER did not investigate reasons for the preference for low atorvastatin doses. It is possible that physicians want to avoid side effects associated with higher doses. Indeed, muscle-related adverse events, cognitive and memory problems, and increase of liver enzymes have been reported to occur more frequently at higher doses [8, 30]. Generally a 5–10 % rate of treatment-associated adverse events is associated with statin use in randomised clinical trials. [16] Under clinical practice conditions, the observed rates appear to be higher, probably owing to the higher proportion of patients with various comorbidities, with complex concomitant medication patterns or further factors that might complicate pharmacotherapy (such as alcohol consumption) [23]. For example, in a study in Boston, 17.4 % of patients reported side effects, and 53.1 % had at least one therapy interruption [38]. Other possible explanations include a lack of awareness/communication of the clinical trial data and the respective guidelines.

Our analysis showed that patients with concomitant CHD and DM were better managed in terms of LDL-C goal attainment, and treated with higher atorvastatin doses compared to patients who had only one of these conditions. This finding is in line with earlier studies in Germany and other countries that showed that patients with cardiovascular conditions are treated more intensively at a later stage of the disease, after complications have occurred [1, 32].

### Limitations

The current study collected data retrospectively at one time point. However, the majority of patients were under maintenance therapy which allows a reliable snapshot of the current situation. In prospective observational studies, the rate of patients with achieved target values usually increases between the inclusion and the follow-up visits. This might be due to the study situation, but also due to the feedback of target values for the individual patient. DISCOVER used various quality measures and plausibility checks, included patients in all regions of Germany, and stipulated the consecutive inclusion of eligible patients at the sites. Site selection focused on those physicians who usually make treatment decisions on statin use. General practitioners/family physicians, internists and cardiologists accounted for 64.7, 26.2 and 6.7 % in our study. The shares of these physician groups among all active German physicians are 42.0, 10.2, and 1.2 %, respectively [3]. As only few inclusion/exclusion criteria applied, typical patients under real life conditions were documented including those with comorbidities and concomitant medication. Notably, in non-participating centers and non-participating patients the situation may be different, as those willing to participate may be more adherent to guideline-oriented therapy compared to those declining. No data on medication adherence to statins were collected [7]; however, use of generic drugs compared to the originals likely plays no major role for adherence [28]. Lastly, the LDL-C levels of patients before the initiation of drug treatment were not known, but would have been of interest to assess the percentage lipid-lowering effect of atorvastatin compared to the situation in the untreated patient.

In conclusion, LDL-C target achievement rates as stipulated by current EAS/ESC guidelines for high-risk individuals under conditions of current clinical practice often were not met despite treatment with the potent lipid-lowering drug atorvastatin. Atorvastatin was prescribed at relatively low doses (mean 28 mg/day). Only one in ten patients on atorvastatin reached the LDL-C target of <70 mg/dL, which is substantially less than rates achieved in other countries. Physicians very often stated that their patients had achieved their individual lipid targets which were in stark contrast to the low achievement rates as evidenced by objective measurements of LDL-C values. Better communication of clinical evidence and the guidelines appears to be needed. Improved lipid management of these high cardiovascular risk patients can be achieved by using higher doses of potent statins and/or combination therapy.

## References

[1] Bestehorn K, Jannowitz C, Karmann B, Pittrow D, Kirch W (2009). Characteristics, management and attainment of lipid target levels in patients enrolled in Disease Management Program versus those in routine care: LUTZ registry. BMC Public Health.

[2] Bestehorn K, Junger C, Smolka W, Gitt AK (2011). Regional differences in the treatment of dyslipidemia in Germany. Dtsch Med Wochenschr.

[3] Bundesärztekammer: Jahresstatistik Ärzte in Deutschland zum 31.12.2014 [annual statistics on physicians in Germany as of 31 Dec 2014]. http://www.bundesaerztekammer.de/ueber-uns/aerztestatistik/aerztestatistik-2014/berufstaetige-aerzte/. Accessed 6 Mar 2016

[4] Castro A, Rosillo SO, Alonso A, Pedersen T (2012). Critical appraisal of trials for validation of ‘risk-guided’ cardiovascular disease prevention therapy. Eur J Prev Cardiol.

[5] Collaborators Cholesterol Treatment Trialists (2012). The effects of lowering LDL cholesterol with statin therapy in people at low risk of vascular disease: meta-analysis of individual data from 27 randomised trials. Lancet.

[6] Davis M, Diamond J, Montgomery D, Krishnan S, Eagle K, Jackson E (2015). Acute coronary syndrome in young women under 55 years of age: clinical characteristics, treatment, and outcomes. Clin Res Cardiol.

[7] De Vera MA, Bhole V, Burns LC, Lacaille D (2014). Impact of statin adherence on cardiovascular disease and mortality outcomes: a systematic review. Br J Clin Pharmacol.

[8] Egan A, Colman E (2011). Weighing the benefits of high-dose simvastatin against the risk of myopathy. N Eng J Med.

[9] Ewen S, Baumgarten T, Rettig-Ewen V, Mahfoud F, Griese-Mammen N, Schulz M (2015). Analyses of drugs stored at home by elderly patients with chronic heart failure. Clin Res Cardiol.

[10] Genser B, Marz W (2006). Low density lipoprotein cholesterol, statins and cardiovascular events: a meta-analysis. Clin Res Cardiol.

[11] Gitt AK, Junger C, Jannowitz C, Karmann B, Senges J, Bestehorn K (2011). Adherence of hospital-based cardiologists to lipid guidelines in patients at high risk for cardiovascular events (2L registry). Clin Res Cardiol.

[12] Gitt AK, Junger C, Smolka W, Bestehorn K (2010). Prevalence and overlap of different lipid abnormalities in statin-treated patients at high cardiovascular risk in clinical practice in Germany. Clin Res Cardiol.

[13] Hiro T, Kimura T, Morimoto T, Miyauchi K, Nakagawa Y, Yamagishi M (2009). Effect of intensive statin therapy on regression of coronary atherosclerosis in patients with acute coronary syndrome: a multicenter randomized trial evaluated by volumetric intravascular ultrasound using pitavastatin versus atorvastatin (JAPAN-ACS [Japan assessment of pitavastatin and atorvastatin in acute coronary syndrome] study). J Am Coll Cardiol.

[14] Jameson K, Zhang Q, Zhao C, Ramey DR, Tershakovec AM, Gutkin SW (2014). Total and low-density lipoprotein cholesterol in high-risk patients treated with atorvastatin monotherapy in the United Kingdom: analysis of a primary-care database. Curr Med Res Opin.

[15] Karlson BW, Palmer MK, Nicholls SJ, Lundman P, Barter PJ (2015). To what extent do high-intensity statins reduce low-density lipoprotein cholesterol in each of the four statin benefit groups identified by the 2013 American College of Cardiology/American Heart Association guidelines? A VOYAGER meta-analysis. Atherosclerosis.

[16] Kashani A, Phillips CO, Foody JM, Wang Y, Mangalmurti S, Ko DT (2006). Risks associated with statin therapy: a systematic overview of randomized clinical trials. Circulation.

[17] Klose G, Schwabe U, Schwabe U, Paffrath D (2014). Lipidsenkende Mittel. Arzneiverordnungs-Report 2014. Aktuelle Daten, Kosten, Trends und Kommentare.

[18] Kotseva K, Wood D, De Bacquer D, De Backer G, Rydén L, Jennings C, Gyberg V, Amouyel P, Bruthans J, Castro Conde A, Cífková R, Deckers JW, De Sutter J, Dilic M, Dolzhenko M, Erglis A, Fras Z, Gaita D, Gotcheva N, Goudevenos J, Heuschmann P, Laucevicius A, Lehto S, Lovic D, Miličić D, Moore D, Nicolaides E, Oganov R, Pajak A, Pogosova N, Reiner Z, Stagmo M, Störk S, Tokgözoğlu L, Vulic D, EUROASPIRE Investigators (2016). EUROASPIRE IV: A European Society of Cardiology survey on the lifestyle, risk factor and therapeutic management of coronary patients from 24 European countries. Eur J Prev Cardiol.

[19] Law MR, Wald NJ, Rudnicka AR (2003). Quantifying effect of statins on low density lipoprotein cholesterol, ischaemic heart disease, and stroke: systematic review and meta-analysis. BMJ.

[20] Lee CW, Kang SJ, Ahn JM, Song HG, Lee JY, Kim WJ (2012). Comparison of effects of atorvastatin (20 mg) versus rosuvastatin (10 mg) therapy on mild coronary atherosclerotic plaques (from the ARTMAP trial). Am J Cardiol.

[21] Maddox TM, Borden WB, Tang F, Virani SS, Oetgen WJ, Mullen JB (2014). Implications of the 2013 ACC/AHA cholesterol guidelines for adults in contemporary cardiovascular practice: insights from the NCDR PINNACLE registry. J Am Coll Cardiol.

[22] Mangiapane S, Busse R (2011). Prescription prevalence and continuing medication use for secondary prevention after myocardial infarction: the reality of care revealed by claims data analysis. Dtsch Arztebl Int.

[23] Maningat P, Breslow JL (2011). Needed: pragmatic clinical trials for statin-intolerant patients. N Engl J Med.

[24] Marrett E, Zhao C, Zhang NJ, Zhang Q, Ramey DR, Tomassini JE (2014). Limitations of real-world treatment with atorvastatin monotherapy for lowering LDL-C in high-risk cardiovascular patients in the US. Vasc Health Risk Manag.

[25] Naci H, Brugts JJ, Fleurence R, Ades AE (2013). Dose-comparative effects of different statins on serum lipid levels: a network meta-analysis of 256,827 individuals in 181 randomized controlled trials. Eur J Prev Cardiol.

[26] Naci H, Brugts JJ, Fleurence R, Tsoi B, Toor H, Ades AE (2013). Comparative benefits of statins in the primary and secondary prevention of major coronary events and all-cause mortality: a network meta-analysis of placebo-controlled and active-comparator trials. Eur J Prev Cardiol.

[27] Nicholls SJ, Ballantyne CM, Barter PJ, Chapman MJ, Erbel RM, Libby P (2011). Effect of two intensive statin regimens on progression of coronary disease. N Engl J Med.

[28] O’Brien EC, McCoy LA, Thomas L, Peterson ED, Wang TY (2015). Patient adherence to generic versus brand statin therapy after acute myocardial infarction: insights from the Can Rapid Stratification of Unstable Angina Patients Suppress Adverse Outcomes with Early Implementation of the American College of Cardiology/American Heart Association Guidelines Registry. Am Heart J.

[29] Palmer MK, Nicholls SJ, Lundman P, Barter PJ, Karlson BW (2013). Achievement of LDL-C goals depends on baseline LDL-C and choice and dose of statin: an analysis from the VOYAGER database. Eur J Prev Cardiol.

[30] Parker BP, Polk DM, Rabdiya V (2010). Changes in memory function and neuronal activation associated with atorvastatin therapy. Pharmacotherapy.

[31] Pittrow D, Pieper L, Klotsche J, Wittchen H (eds) (2007) DETECT. Ergebnisse einer klinisch-epidemiologischen Querschnitts- und Verlaufsstudie mit 50.000 Patienten in 3.000 Hausarztpraxen. 1st edn. Elsevier, Munich

[32] Pittrow D, Stalla GK, Zeiher AM, Silber S, Marz W, Pieper L (2006). Prevalence, drug treatment and metabolic control of diabetes mellitus in primary care. Med Klin (Munich).

[33] Reiner Z, Catapano AL, De Backer G, Graham I, Taskinen MR, Wiklund O (2011). ESC/EAS Guidelines for the management of dyslipidaemias: the Task Force for the management of dyslipidaemias of the European Society of Cardiology (ESC) and the European Atherosclerosis Society (EAS). Eur Heart J.

[34] Schoen MW, Salas J, Scherrer JF, Buckhold FR (2015). Cholesterol treatment and changes in guidelines in an academic medical practice. Am J Med.

[35] Setia S, Fung SS, Waters DD (2015). Doctors’ knowledge, attitudes, and compliance with 2013 ACC/AHA guidelines for prevention of atherosclerotic cardiovascular disease in Singapore. Vasc Health Risk Manag.

[36] Stone NJ, Robinson JG, Lichtenstein AH, Bairey Merz CN, Blum CB, Eckel RH (2013). ACC/AHA guideline on the treatment of blood cholesterol to reduce atherosclerotic cardiovascular risk in adults: a report of the American College of Cardiology/American Heart Association Task Force on Practice Guidelines. J Am Coll Cardiol.

[37] Werner C, Filmer A, Fritsch M, Groenewold S, Graber S, Bohm M (2014). Risk prediction with triglycerides in patients with stable coronary disease on statin treatment. Clin Res Cardiol.

[38] Zhang H, Plutzky J, Skentzos S, Morrison F, Mar P, Shubina M (2013). Discontinuation of statins in routine care settings: a cohort study. Ann Intern Med.

